# Functional constraints and evolutionary dynamics of the repeats in the rDNA internal transcribed spacer 2 of members of the *Anopheles barbirostris* group

**DOI:** 10.1186/1756-3305-7-106

**Published:** 2014-03-19

**Authors:** Claudia Caterina Paredes-Esquivel, Harold Townson

**Affiliations:** 1 Department of Vector Biology, Liverpool School of Tropical Medicine, Pembroke Place, Liverpool, L3 5QA United Kingdom; 2 Current address: Laboratory of Zoology. University of the Balearic Islands, Ctra de Valldemossa Km 7.5, 07122 Palma de Mallorca, Spain

**Keywords:** *Anopheles barbirostris*, ITS2, Secondary structure, Repeat, DNA insertion

## Abstract

**Background:**

The *Anopheles barbirostris* group is widely distributed in Southeast Asia. Although seven species have been formally described, a molecular analysis of the rDNA ITS2 and the mitochondrial cytochrome oxidase I gene suggests that the group includes species that are morphologically very similar or identical.

We have previously shown that species in the *Anopheles barbirostris* Subgroup have an exceptionally large ITS2 (>1.5 kb), greater than in any other *Anopheline* group. However, the molecular processes responsible for generating such a large ITS2 have not previously been explored.

**Methods:**

To determine the processes by which this large ITS2 is generated, we examined the sequence and secondary structure of the ITS2 of 51 specimens from five species of the *Anopheles barbirostris* Subgroup. These include the anthropophilic species *An. campestris* and three morphospecies of the Barbirostris Complex: *An. vanderwulpi*, *An. barbirostris* I and III, together with a previously undescribed member of this group (Clade IV).

**Results and conclusions:**

All the specimens were found to have an ITS2 greater than 1.5 kb in length. The possibility that the spacer sequences amplified were pseudogenes was examined and discarded. The large size of ITS2 in the species studied is due to the presence of internal repeats of approximately 110 bp in length, confined to the central region of the spacer. Repeats varied markedly between the species examined, with respect to their organization, number and sequence similarity. The nucleotide diversity increased in direct relation to size variation and the presence of non-repeated elements.

A secondary structure analysis showed that the repeats form hairpin structures with a wide range of free energy values. These hairpin structures are known to facilitate the subsequent processing of mature rRNA. An analysis of the repeats from the different species suggests they originate from a common ancestor, with the repeats appearing before speciation of the Barbirostris Group.

## Background

In the ribosomal genome, the first and second internal transcribed spacers (ITS1 and ITS2) are located between the genes coding for 18S, 5.8S and 28S in the ribosomal DNA cluster. Although spacer sequences are not present in the mature ribosomal RNA molecules, they mediate the cleavage of the great RNA precursor [1] and form stable secondary structures [2]. There is evidence that the deletion of ITS2 affects the maturation of both the small and large subunit rRNA [3]. Whilst many mutations become fixed in a population through natural selection and genetic drift, the non-coding spacer regions in ITS2 evolve through a pattern of concerted evolution [4]. Typically, ITS2 exhibits a low level of sequence variation within species and a high level of divergence between species. It is this feature that renders the region useful to infer phylogenetic relationships in different taxa, including insects [5]. In *Anopheles* species, ITS2 has proved valuable for distinguishing members of sibling species complexes [6-8] and is the most commonly used marker for the design of species-specific PCR primers for species discrimination [9].

The *Anopheles barbirostris* group is distributed in the oriental region [10]. Some of its members have been implicated in malaria and filariasis transmission in Southeast Asia [11-13]. Seven species have been formerly described: *An. barbirostris, An. campestris, An. donaldi, An. pollicaris, An. franciscoi, An. hodgkini*[14] and *An. vanderwulpi*[15], the latter species being found in sympatry with *An. barbirostris sensu stricto* in Eastern Java. *Anopheles campestris* is considered the most anthropophilic of these species. Variations in habitat, resting behaviour and feeding preferences have been reported in the *Anopheles barbirostris* complex [16-18]. A molecular analysis of ITS2 and the cytochrome oxidase I (*COI*) gene region confirmed that the morphospecies *An. barbirostris* comprises at least three morphologically identical species, including *An. vanderwulpi*. In addition, one species, (Clade IV of Paredes-Esquivel, 2009), shares morphological characters with *An. barbirostris* and *An. campestris*.

All species in the Barbirostris Subgroup have a large ITS2 (>1.5 kb). The size varies between Clades, namely: Clade I, 1545 bp; Clade II, 1727 bp; Clade III, 1730 bp; Clade IV, 1583 bp and *An. campestris*, 1519 bp. ITS2 sequences of considerable size have been documented in other *Anopheles* species. *Anopheles crucians* species B has an ITS2 of 1012 bp [8]. This length was the result of a complex array of repeats in different combinations. In *Anopheles beklemishevi*, a member of the Maculipennis Group, ITS2 comprises 638 bp, including two repeats of about 140 bp of similar though not identical sequence [19]. Similarly, *An. fluminensis* from Bolivia has been reported to have three repeats of 125 bp each within an ITS2 of 596 bp [20]. In *Anopheles* species of the genus *Cellia*[21], a series of internal repeats has led to a large ITS1. ITS2 of considerable length have also been seen in other insect species [8,22,23].

In this paper we establish that the outstanding size of ITS2 in the Barbirostris Subgroup is due to the presence of DNA insertions that occur as repeated units located in the central region of the internal transcribed spacer. These vary in copy numbers and organization. To determine whether these mutations affect the stability of the ITS2 and to discard the possibility that they were pseudogenes, i.e. non-functional relatives of genes, we analysed their secondary structure. We have carried out a comprehensive analysis of these repeated elements and discuss their origin in relation to the evolutionary history of the Barbirostris Group members.

## Methods

The species examined were members of the Barbirostris Group studied by Paredes-Esquivel *et al*. (2009): *Anopheles barbirostris* clade I collected from Kalimantan (Indonesia) and Mae Hong Son (Thailand); *An. vanderwulpi*[15] from the island of Sumatra (Indonesia); *An. barbirostris* clade III, collected from Mae Hong Son, Sa Kaeo, Tak and Kanchanaburi (Thailand); an unknown species clade IV, with mixed characters between *An. barbirostris* and *An. campestris*[10] found in Sumatra and Trat and Sa Kaeo (Thailand) and finally *An. campestris* collected in Sa Kaeo. These species were identified based on the analysis of the *COI*, ITS2, combined with morphological examination of adult specimens. GenBank accession numbers, sequence sizes and place of collection are summarized in Table 1. Detailed DNA extraction and amplification procedures are described in Paredes-Esquivel *et al.* (2009).

**Table 1:** List of specimens and size of ITS2 (bp)

	**Specimen**	**GenBank Acc N°**	**ITS2 size(bp)**	**Locality**
	k2	EU812759	1545	Kalimantan (Indonesia)
	k3	EU812760	1544	“
*An. barbirostris*	th1.1	EU812761	1544	Mae Hong Son (Thailand)
Clade I	th1.3	EU812764	1542	“
	th1.4	EU812762	1542	“
	th1.7	EU812763	1542	“
	l12	EU812766	1727	Sumatra (Indonesia)
*An. vanderwulpi*	l13	EU812765	1727	“
	l15	EU812767	1727	“
	l33	EU812768	1727	“
	th39.3	EU812781	1732	Mae Hong Son (Thailand)
	th1.6	EU812776	1732	“
	th1.2	EU812775	1733	“
	th1.8	EU812782	1733	“
	th1.9	EU812780	1732	“
	th1.10	EU812779	1732	“
	bsk33	EU812769	1733	Sa Kaeo (Thailand)
	bsk5	EU812790	1733	“
*An. barbirostris*	S17.1	EU812770	1733	“
Clade III	S11.2	EU812778	1733	“
	S24.3	EU812789	1731	“
	ta10	EU812785	1733	Tak (Thailand)
	ta19	EU812774	1733	“
	ta21	EU812771	1734	“
	ta22	EU812772	1733	“
	ta23	EU812784	1733	“
	ta24	EU812783	1732	“
	kh3	EU812787	1732	Kanchanaburi (Thailand)
	kh4	EU812786	1733	“
	kh7	EU812773	1732	“
	kh9	EU812777	1732	“
	kh10	EU812788	1732	“
	l14	EU812791	1581	Sumatra (Indonesia)
	btr7	EU812795	1584	Trat (Thailand)
	btr8	EU812798	1584	“
	btr10	EU812796	1582	“
	btr11	EU812799	1584	“
	btr16	EU812792	1584	“
	btr17	EU812801	1583	“
	btr18	EU812793	1584	“
Unknown species	btr19	EU812802	1585	“
	btr22	EU812797	1585	“
	btr23	EU812800	1584	“
	ctr2	EU812804	1585	“
	ctr4	EU812803	1585	“
	T35.1	EU812807	1584	“
	T35.2	EU812806	1585	“
	bsk3	EU812794	1585	Sa Kaeo (Thailand)
	S24.1	EU812805	1585	“
*Anopheles campestris*	csk10	EU812808	1519	Sa Kaeo (Thailand)
	bsk34	EU812809	1519	“

Sequence alignment was complicated by the presence of internal repeats. BioEdit v 5.0.6. (Hall 1999) was used to align sequences manually and to determine GC content and length. Boundaries of the ITS2 region were identified in comparison to sequences from *Anopheles gambiae* (GenBank accession number X67157.1) [24]. When the regions containing the repeats were removed, sequences could be aligned with ClustalW [25]. The substitutional rate in the conserved 5,8S and 28 S regions was analysed in detail to discriminate functional genes from pseudogenes. Repeats were found using the Tandem repeats finder program: http://tandem.bu.edu/trf/trf.html, although an exhaustive visual examination was also required. Nucleotide diversity (π) was calculated with program DNASP5.101 [26] to determine the degree of polymorphism within each type of repeat. This is defined as the average number of pairwise nucleotide substitutions divided by the length of the sequence (π = П/*L*) [27].

Sequences were folded using RNA Folding Form (version 2.3 energies), from the Mfold web server [28], located at: http://mfold.rna.albany.edu/?q=mfold/RNA-Folding-Form2.3. Default parameters (37°C with 5% suboptimal folding) were used to fold sequences. This program provides with several tentative secondary structures for a wide range of free energy values. Only the ITS2 region was included in the analysis.

BLAST searches were carried out to identify sequence similarities. A Bayesian analysis using Mr Bayes 3.2 [29] was carried out to determine phylogenetic relationships among repeats. For this analysis, the Monte Carlo (MCMC) chain length involved 500,000 generations, with trees sampled every 100 generations. The analysis was repeated twice to confirm topologies. Posterior probabilities were employed to test statistical support for clades. Trees were visualized using the FigTree program http://tree.bio.ed.ac.uk/software/figtree/).

## Results

### Sequence analysis and occurrence of repeat elements

The fifty-one specimens identified as five members of the Barbirostris Group included 47 haplotypes, thus the majority of haplotypes contained a single specimen. The G + C content varied between 52.3% and 55.9%, similar to that found in other *Anopheles* species. All specimens showed an exceptionally large ITS2 amplicon. The size of ITS2 in the members of the Barbirostris Group examined varied markedly in size: Clade I c. 1543 bp., Clade II 1727 bp, Clade-III had a similar ITS2 size ~ 1730 bp but differed in 30 fixed substitutions. In *Anopheles campestris* ITS2 was 1519 bp long whilst in the unnamed member of the Barbirostris Group, Clade IV, ITS2 was 1583 bp. (Table 1).

**Figure 1:**
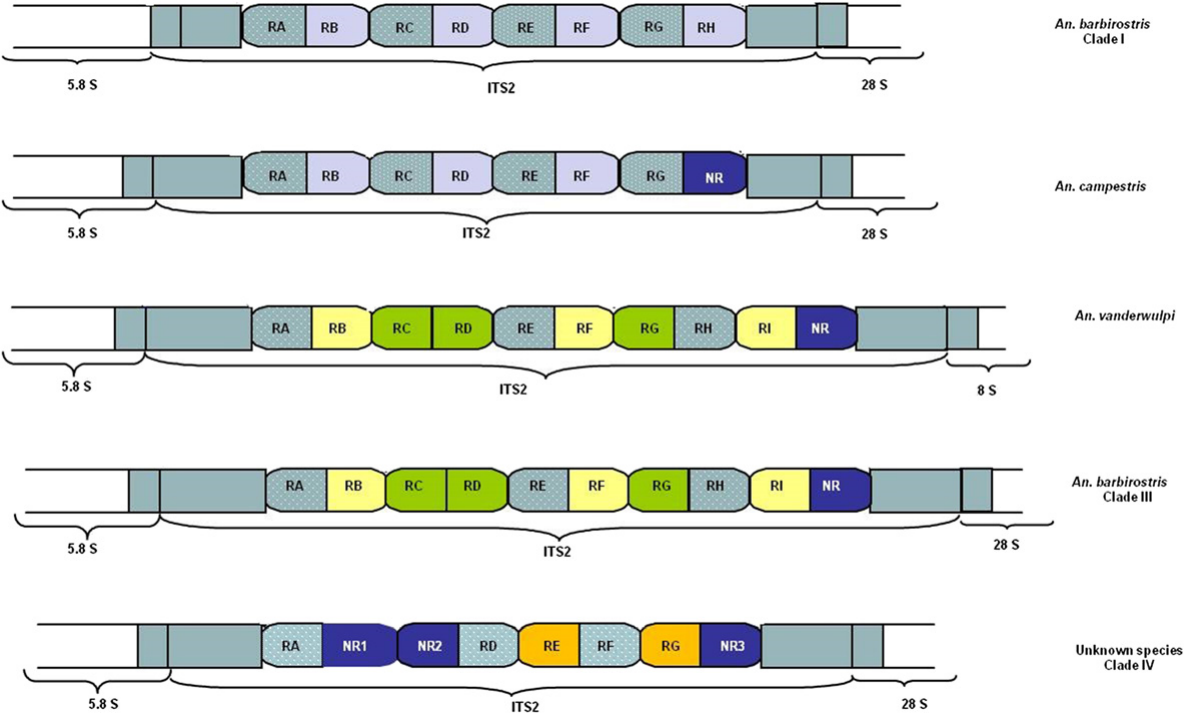
**Showing location of repeats in ITS2 in five members of the Barbirostris group.** Repeats (R) are symbolized with letters indicating position from 5′ end of ITS2 (A being closest). Non-repeated elements (NR) are located at 3′ end. Similar filled patterns represent similar sequences.

The exceptional length of the ITS2 region was found to be due to the presence of repeated elements, located at the centre of the ITS2, occupying 55% to 61% of the total length of the spacer. The number and organization of the repeats varied between species (Figure 1 and Table 2). In Clade I, eight repeats, organized in two groups comprising four repeats each, were present. There was no length variation in these repeats, being 112 and 108 bp long for types 1 and 2, respectively. Repeats in *Anopheles campestris* were arranged in a similar manner, except that the last repeat at the 3′ end turned into a non-repeated element. The presence of a single insertion/deletion event resulted in length variation in these repeats (Table 2). *Anopheles vanderwulpi* and Clade III showed a similar arrangement of nine repeated elements of three types. In both of these, sequences differed by several nucleotides. Non-repeated elements were also present in these species. Finally, the unknown species of the Barbirostris Group (Clade IV) contained five repeats of two types and varied sizes and three non-repeated elements (Figure 1). Most indels were located at the 3′ end of the repeat sequences in species where indels were found.

**Table 2:** Nucleotide diversity and sequence variation in repeats

**Repeats type**	**Repeats**	**N° of SS**	**π**	N° sites compared*	**Sequence lengths**
Clade I type 1	A, C, E, G	2	0.01	112	112
Clade I type 2	D, B, F, H	5	0.02	108	108
*An. campestris* type 1	A, C, E, G	4	0.02273	110	110 (G), 111 (A,C,E)
*An. campestris* type 2	D, B, F	9	0.05	112	112 (F), 113 (D,B)
*An. vanderwulpi* type 1	A, E, H	11	0.08	103	103 (E), 108 (H), 117 (A)
*An. vanderwulpi* type 2	C, D, G	6	0.04	102	102 (D), 105 (G), 107 (C)
*An. vanderwulpi* type 3	B, F, I	6	0.04	98	99 (B,F), 110 (I)
Clade III type 1	A, E, H	17	0.11	107	107 (E), 113 (H), 116 (A)
Clade III type 2	C, D, G	7	0.05	101	103 (D,G), 105 (C)
Clade III type 3	B, F, I	7	0.05	98	99 (B), 101 (F), 113 (I)
Clade IV type 1	A, D, F	5	0.04	93	97 (F), 98 (D), 116 (A)
Clade IV type 2	E, G	16	0.14	114	114 (E), 119 (G)

*SS:* segregating sites.

*excluding sites with gaps/missing data.

Table 2 shows the nucleotide diversity of the different type of repeats found in Barbirostris Group members. The nucleotide diversity π increased in direct relation to size variation and the presence of non-repeated elements. The lowest π values were observed in repeats of Clade I and *An. campestris*, whereas the unknown species Clade IV showed a high degree of polymorphism, particularly in type 2 repeats.

**Figure 2:**
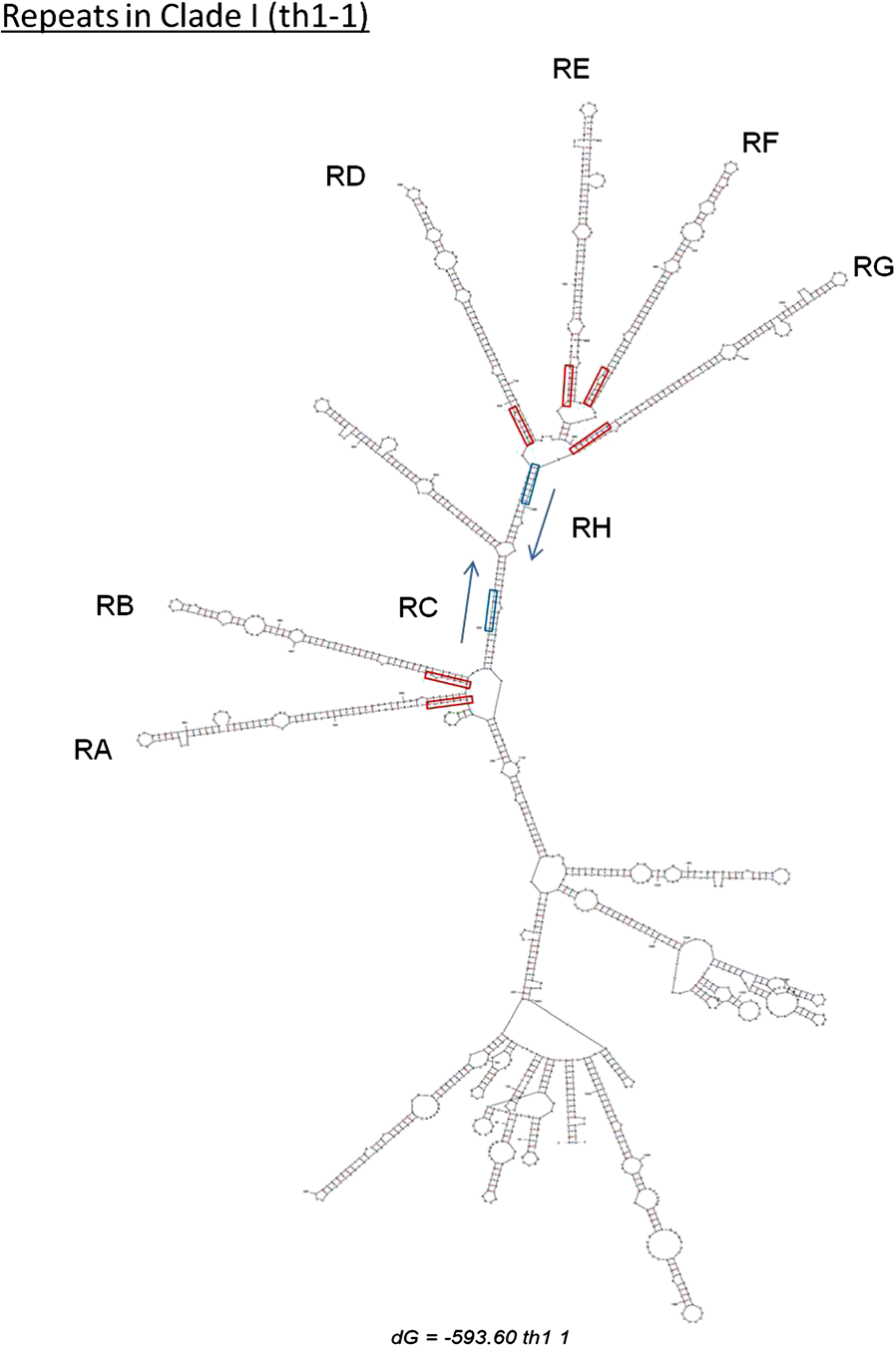
**Secondary structure reconstruction of ITS2 in Clade-I of the *An*. *barbirostris *subgroup.** The motif *GGGTGTG* is represented with a rectangle. Each one of the repeats A, B, D, E, F and G form hairpin structures.

A *GGGTGTG* motif occurred at the 5′ end of most repeats. Although this motif was common to all species, in some cases variations of this motif were also observed (*GGGTGGT* in *An. campestris*, *GGGTGGG* in clade-I and *An. vanderwulpi*, GGGTGCG and TGGTGTG in the unknown member of the Barbirostris Subgroup). Using default parameters, most repeats formed long hairpin structures with a wide range of energy values. Results varied only in the number of hairpins that repeats formed, these being more evident in Clade I, Clade III and in *An. campestris*. At the base of the hairpin stem there was a motif *GGGTGTG* (or similar) at one side and a palindromic sequence on the other side of the stem. As an example Figure 2 shows the disposition of the hairpins in Clade I, at the lowest free energy value. Interestingly in this species each hairpin has a similar structure with three internal loops located close to the top of the hairpin (Figure 2). Stable RNA secondary structures are characterised by having low energy values. Energy values ranged from 593.6 to 629.4. Nevertheless, repeats formed at least 3 hairpin structures in all 25 resultant secondary structures, the only difference being the number of hairpins formed.

## Discussion

Among members of the Barbirostris Group, *An. barbirostris* Clade III has the largest ITS2 recorded so far in *Anopheles* species (~1730 bp). The large size of ITS2 in members of the Barbirostris Group (>1.5 kb) results from the presence of DNA insertions, comprising tandem-repeated elements of c.100 bp located in the centre of the spacer region. In the genus *Anopheles* the ITS2 is characterized by a very low level of polymorphism between species, even between cryptic species [24]. Despite reports of intra-specific and intra-individual variation [21], ITS2 is still widely used to infer phylogenetic relations among species [30,31]. In the Barbirostris Group, the presence of repeats did not affect tree topology, and this was consistent with results from COI analysis (10).

Given the high rate of substitution, we examined the possibility that the ITS2 of members of the Barbirostris Group were pseudogenes. This hypothesis was discarded for the following reasons: 1) There was a low rate of substitution in the flanking 5.8 and 28S regions which were amplified, these comprise 90 nucleotides of the alignment; 2) Hairpin structures were formed for all species in high number, at the lowest free energy values. Pseudogenes in ribosomal spacers tend to accumulate random substitutions at high rates, whereas functional ITS regions show many hairpins with compact stable secondary structures [32]. Even when a point mutation was observed, it can be assumed that the structural integrity was not affected by the insertion of long repetitive elements, as large spacers seem to be less affected by mutations than shorter ones [33].

### Functional constraints on repeats

Long ITS2 regions have been found in distant taxa including oomycetes [34] and amphipods [35]. They have also been reported in several arthropod species, including rhipicephaline ticks [23] and insects of various orders [36,37]. Repetitive DNA regions of considerable size have also been documented in the ITS2 of other *Anopheles* species [8,19,20]. Little has been written to explain the origin and function of repeated elements in ribosomal spacer regions. Secondary structure reconstructions show that repeats found in members of the Barbirostris Group form hairpin structures at a wide range of energy values. Since the Mfold server does not produce a single ‘correct folding’, many configurations obtained with the minimum free energy values were examined. It is known that hairpin structures are related to functional ITS regions [32], although their functionality has not been fully elucidated. As stated by Paskewitz *et al.*[24] in their pioneering study of the secondary structure of ITS1 in the *Anopheles gambiae* complex, conservation of hairpin structures in a wide range of energy values may indicate the conservation of functional constraints, possibly related to the maturation of ribosomal subunits. Long stem-loop secondary structures are also formed by the repeats found in the ITS1 of members of the *An. punctulatus* group [21]. However, the repeats found in the Barbirostris group showed no similarity to repeats in *An. punctulatus* or *An. gambiae.* Furthermore, a Blast search failed to reveal any similar sequence in GenBank. A possible explanation for this is that similarities to other insertional elements, e.g. transposable elements, may be obscured by the high rate of substitutions present in this spacer, as has been observed in other taxa [36].

Variation in other regions of the ribosomal gene has been reported as a result of adaptations to changes in the environment. Thus in the parthenogenetic *Daphnia pulex,* longer IGS regions provide these organisms with the plasticity required to adapt to different environments [38]. In Bryophytes, repetitive sequences in the rDNA appear to result from exposure to heavy metals. Whether insertion elements or adaptation to new environments affect the ITS2 region of members of the Barbirostris Group has yet to be determined.

ITS2 in members of the Barbirostris Group is subject to a high rate of evolutionary change. We observed a high rate of substitution, even at an intraspecific level and incomplete homogenization of repeats. Nevertheless, when comparing the rate of substitutions at an interspecies level, ITS2 seems to evolve through a pattern of concerted evolution. Accordingly, repeated elements found were imperfect copies of an original one. This has also been reported in the long spacers of other taxa [22,39,40] and seems to be the result of replication slippage events [23].

Hairpin structures appear to facilitate subsequent processing [26]. Thus rRNA processing in yeast requires sequences and/or higher order structures within ITS2, an example of which would be the extensive folding of ITS2 sequences, bringing into juxtaposition those regions of mature 5.8S and 25S rRNA that must interact during processing. Thus rRNA processing requires sequences and/or higher order structures within ITS2. One example may be the extensive folding of the ITS2 sequences (see Figure 2), bringing into juxtaposition those regions in mature 5.8S and 28S rRNA that must interact but that are at a distance in the primary sequence.

Nucleotide diversity varied considerably among repeat types. It was higher in repeats from *An.vanderwulpi*, Clade III and Clade IV, where non-repeated elements were observed and the length of the repeats varied considerably. On the other hand, the degree of polymorphism was much lower in *An.campestris* and particularly in Clade I, where indels were absent. Concerted evolution is a potent force in the formation of ITS2, but there are cases where concerted evolution is incomplete, as seen in *Anopheles longirostris* from Papua New Guinea [33]. It is conceivable that concerted evolution is more effective in Clade I, leading to the stabilization of the repeats. This species is to date the most widely distributed member of the *Anopheles barbirostris* complex, having been reported in Thailand, Borneo (Indonesia), Vietnam [10] and in the islands of Sulawesi and Java, in the Indonesian archipelago [41]. Indels did not affect hairpin structures, as these were located at the 3′ end, outside of hairpin structures.

We postulate that repeat elements were present in the common ancestor of the species of the Barbirostris Group, before members of the Group underwent subsequent speciation. This conclusion is based on two principal facts; firstly repeats in all species were flanked by a common motif (*GGGTGTG* and variants) and more importantly, the Bayesian analysis shows that type I repeats present in *An. campestris* form a monophyletic clade with their homologous repeats in Clade I.

## Conclusions

We conclude that repeat elements were present in the common ancestor of species of the Barbirostris Group, before members of the Group underwent subsequent speciation. This conclusion is based on two principal observations; firstly repeats in all species are flanked by a common motif (GGGTGTG and variants) and more importantly, the Bayesian analysis shows that type I repeats present in An. campestris form a monophyletic clade with their homologous repeats in Clade I. Repeats form hairpin structures that are linked to the functional constraints on the internal transcribed spacer, since they are known to facilitate the processing of mature rRNA.

## Competing interests

The authors declare that they have no competing interests.

## Authors’ contributions

CPE carried out the molecular studies and prepared draft Figures and Tables. HT assisted in the analysis of the data and their presentation. CPE provided an initial draft of the text. HT and CPE contributed equally to the final article and both authors read and approved the final manuscript.
